# Open Air and Baths

**Published:** 1919-02

**Authors:** 


### Open Air and Baths.

Physicians have long recognized the value of air and cleanliness in the treatment of most diseases, but many of them have not had the courage to prescribe and demand it of patients as they should do. Not only that, but they have known that plenty of fresh, pure air by day and night, and baths, external and internal, will prevent most of the ills to which humanity is susceptible. There is really not much reason why any normal child, properly reared, kept in physical trim, fed a suitable diet, trained to bathe as punctiliously as he eats and made to breathe wholesome air, should not be able to resist all the disease germs afloat, not only in childhood, but to a ripe old age. Nine-tenths of the diseases are due to indifference, carelessness, or ignorance.

Most of the diseases yield to mild treatments, if taken in time, and if accompanied by plenty of air and a thorough cleaning of the body, inside and out. It has been found that nothing else helps an influenza victim so much as all the air that can possibly be given. In a test case 100 sailors, men of good physique and training, volunteered to be subjected to influenza in every way it was thought it could be conveyed. Every effort known to medical men was made to give them the disease, but the men were given all the open air and that careful bathing to which sailors are accustomed at most times, and not a man of the hundred took the influenza.

In every test where the open-air and bath treatment has been given a thorough trial the results have not only been satisfactory, they have been marvelous, and that too with the use of very little medicine. How long will it be before physicians will have the courage to insist that patients take air and water just as they would take other medicines—for these are after all nature's best remedies.

